# Tumor Biology: Is It Time to Redefine Unresectability? An Extraordinary Case of Gastroesophageal Junctional Adenocarcinoma

**DOI:** 10.7759/cureus.420

**Published:** 2015-12-21

**Authors:** Andy Gaya, Alex Giakoustidis, Mark Winslet, Satvinder Mudan

**Affiliations:** 1 London Oncology Clinic, Guy's and St. Thomas' NHS Foundation Trust; 2 Department of HPB Surgery, Royal London Hospital; 3 Department of Surgery, Royal Free Hospital; 4 Department of Academic Surgery, The Royal Marsden Hospital

**Keywords:** esophageal cancer, gastric cancer, radiotherapy, appleby procedure, surgical resection, surgical technique, chemotherapy, immunotherapy

## Abstract

Background: Disease assessment based on measurements of size and anatomic involvement have historically been central to surgical strategy. We propose this to be an outdated concept, which should be replaced by a deeper understanding of tumor biology and careful treatment planning.

Report of case: A 34-year-old male was diagnosed with a Siewert Type 3 locally advanced cancer of the gastroesophageal junction, involving the coeliac axis and the superior mesenteric artery (SMA). He was treated with neoadjuvant chemotherapy, followed by chemoradiation, and then proceeded to surgery, at which time the tumor was judged unresectable. After extensive planning, a further surgery was attempted - an extended gastrectomy with distal esophagectomy, left hepatectomy, and splenectomy were performed. Additionally, the coeliac axis and the SMA were excised, followed by reconstruction of the hepatic artery and the SMA with grafts. Adjuvant chemotherapy was administered, and the patient is recurrence-free after five years follow-up.

Conclusion: This case highlights the importance of the distinction between resectability and operability, and that patient treatment should be tailored and individualised based on the response to treatment, comorbidities, and underlying tumor biology.

## Introduction

Over the past few decades, there has been a dramatic rise in the incidence of adenocarcinoma of the gastroesophageal junction (GEJ) in Western countries, including the UK [1]. Radical surgical excision is the current standard of care [2-3]. Patients diagnosed with locally advanced tumors, which include those with bulky primaries (≥ T3), locoregional lymph node involvement, or where a resection margin is felt threatened, are usually referred for preoperative chemoradiotherapy (CROSS trial approach) or perioperative chemotherapy (MAGIC trial approach) after evaluation by a multidisciplinary team. The current staging system of GEJ tumours includes TNM, grade, vascular invasion, and potential R0/R1/R2 resection margins. Increasingly, an understanding of the genetic basis of cancer and of the host’s immune reactivity may permit the delineation of groups of patients likely to have a better outcome and thus be suitable for more aggressive therapy.

Resectability is determined on a purely technical basis and will depend on the skill and experience of the surgeon. On the other hand, operability will, in addition to resectability, require a consideration of the benefits and risks to the patient, taking into account the age and comorbidities, as to whether a major surgical procedure with its attendant morbidity is justified. We illustrate the difference between unresectability and inoperability by presenting the case of a patient with adenocarcinoma of GEJ, which was initially considered inoperable.

## Case presentation

Informed patient consent was obtained at the time of treatment. No identifying patient information is contained in this paper.

A 34-year-old physically fit male with no comorbidities or family history of cancer presented with resistant dyspepsia and was diagnosed with a locally advanced Type III GEJ cancer extending from 37 cm from the incisors, down onto the lesser curve of the stomach at the level of the incisura. Pathology was a moderately to poorly differentiated adenocarcinoma, HER2 negative. The patient's tumor was considered unresectable at presentation, and he underwent five cycles of neoadjuvant EOX chemotherapy (epirubicin, oxaliplatin, capecitabine) resulting in RECIST stable disease on CT. Extended neoadjuvant therapy was administered due to the locally advanced nature of the tumor. As it was still considered inoperable, consolidation radical chemoradiation to a dose of 50 Gy in 25 fractions with concomitant cisplatin and 5FU was subsequently delivered to the lower esophagus and proximal stomach, leading to significant regression demonstrable at diagnostic laparoscopy. Thereafter, an attempted resection was performed. However, at laparotomy, he was found to have gross disease invading the whole lesser omentum up towards the porta hepatis, with encasement of the left gastric artery and the origin of the coeliac axis as well as extensive serosal disease within the lesser sac and SMA origin. The dimensions of the portion of tumor involving the descending aorta were 3.6 x 2.8 cm.

**Figure 1:**
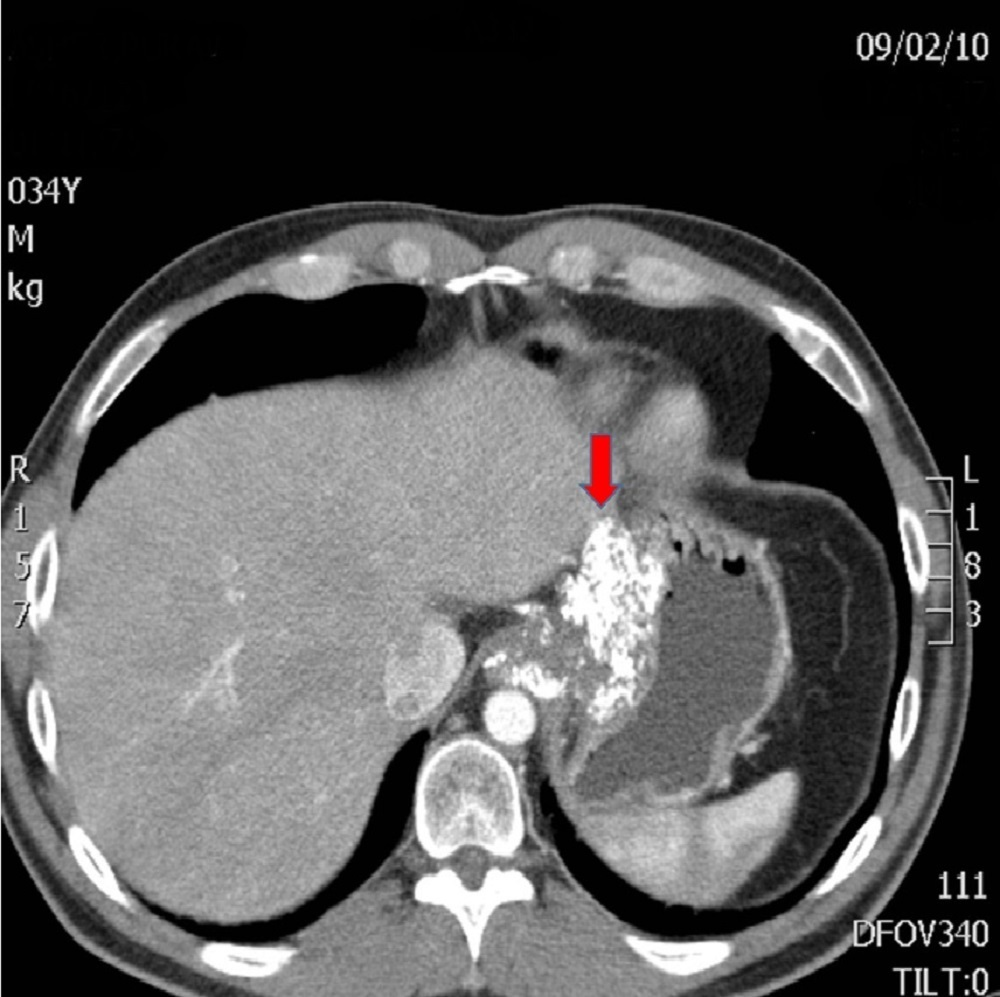
CT and MRI Preoperative Images Axial CT - loss of fat plane between stomach and left lobe of liver indicating possible infiltration by tumor (arrow)

**Figure 2:**
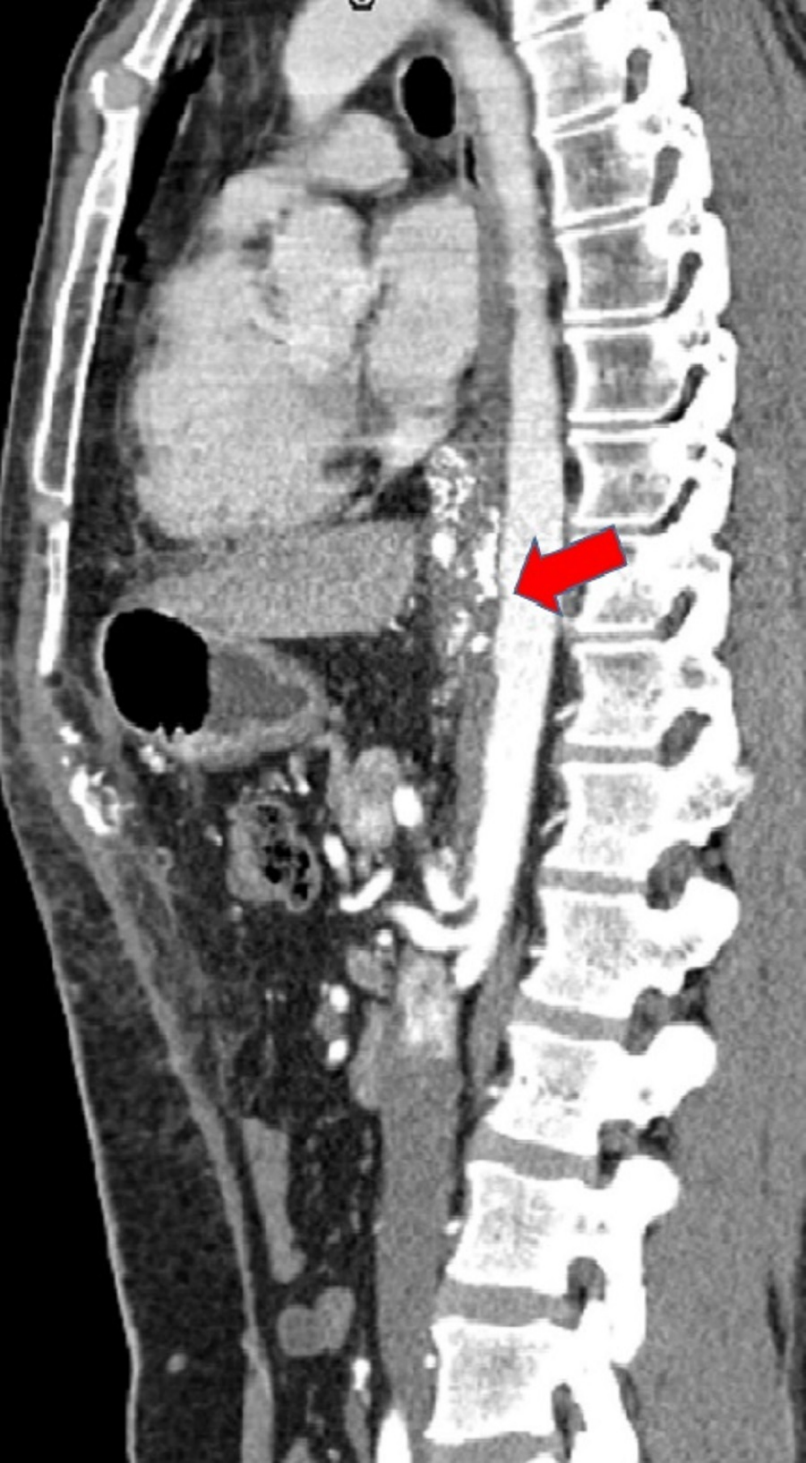
CT and MRI Preoperative Images Sagittal CT - Suspicious of aortic and celiac axis invasion (arrow) - loss of plane between tumor and descending aorta

**Figure 3:**
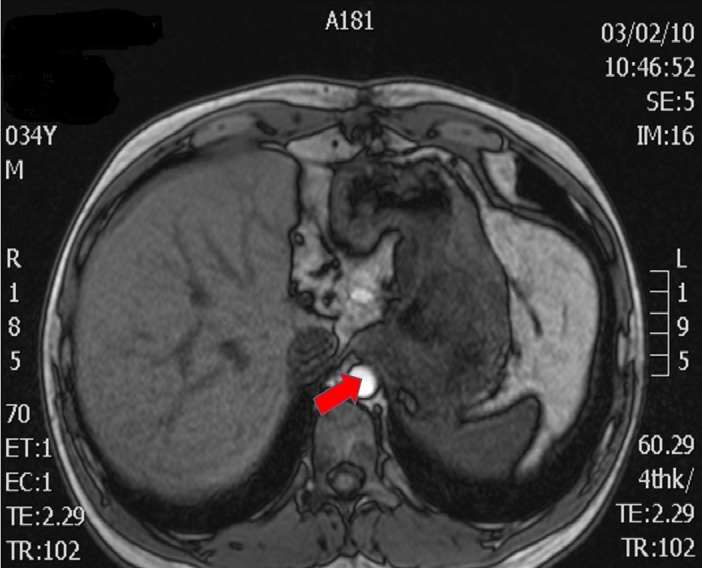
CT and MRI Preoperative Images Axial MRI - Proximity to and potential invasion of descending aorta (arrow)

**Figure 4:**
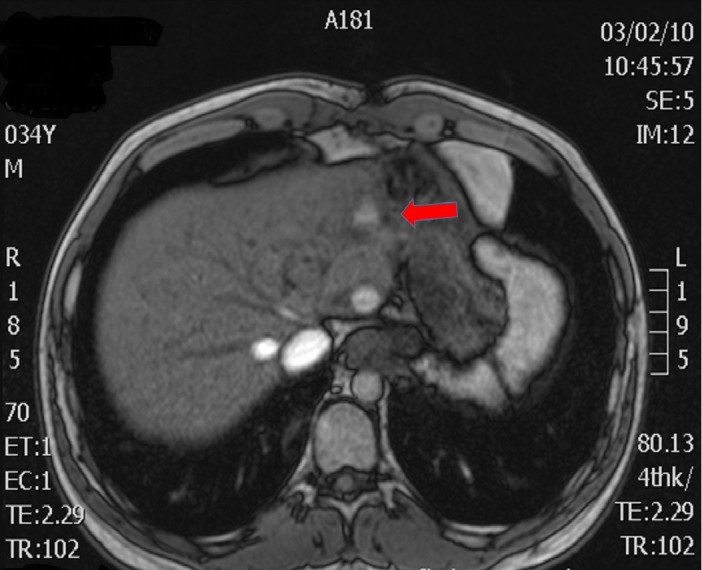
CT and MRI Preoperative Images Axial MRI - Attachment to left liver (arrow)

**Figure 5:**
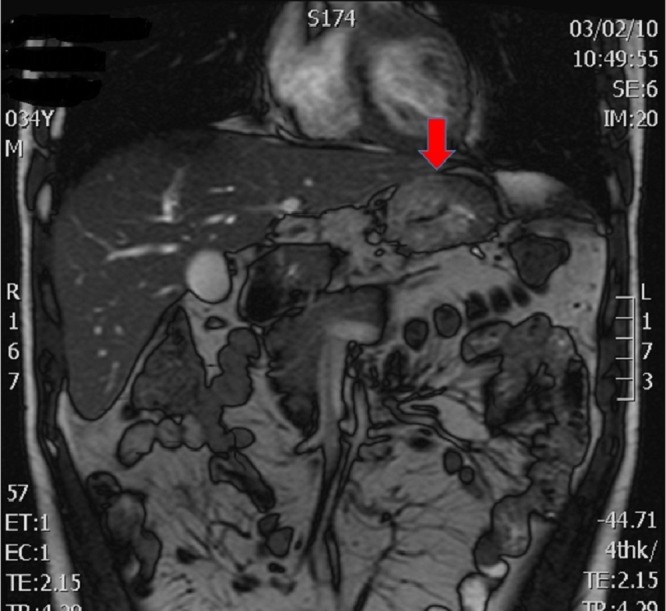
CT and MRI Preoperative Images Coronal MRI - arrow shows primary gastric tumor adjacent to left lobe of liver.

The patient underwent further surgical assessment as, a year after initial diagnosis and six months following completion of chemoradiotherapy, there was no evidence of metastatic disease and the tumor had remained stable on imaging, thereby demonstrating a more indolent tumor biology (Figures 1-5). Six months passed due to the patient obtaining second surgical and oncological opinions, vascular and cardiothoracic opinions, and to undertake preoperative workup and optimisation of cardiorespiratory fitness using cardiopulmonary exercise testing (CPEX) and an intensive prehabilitation exercise regime. A decision was then made with the patient to undertake a further attempt at resection.

A left thoracoabdominal incision was performed with a division of the diaphragm down to the aortic hiatus. The tumor was localized to the GEJ, invading the esophageal hiatus and left crus of the diaphragm. The left lateral segment of the liver was adherent to the stomach and could not be separated, with the tumor extending posteriorly and inferiorly toward the coeliac axis and SMA. The mid-thoracic and abdominal aorta were skeletonized and the visceral arterial branches exposed. The resection included an en-bloc excision of the lower part of the esophagus, the entire stomach, greater omentum, spleen, and the connective tissue of the lesser curve as well as a total bursectomy, including the anterior capsule of the pancreas and the left lateral segment of the liver in order to ensure tumor-free margins. After administration of bolus intravenous heparin, the coeliac axis, and the SMA were ligated at the origin and excised with the adjacent tissue specimen. The hepatic artery and the SMA were revascularised with a bifurcated Dacron graft. Gastrointestinal continuity was restored with a Roux-en-Y esophagojejunal stapled anastomosis at the level of the tracheal bifurcation. Fiducial markers were placed in the tumor bed at sites felt most likely to have a positive margin, in order to guide potential stereotactic radiotherapy in the postoperative period. 

Histopathologic examination of the surgical specimen confirmed a poorly differentiated mucinous adenocarcinoma of the stomach with signet-ring morphology arising from the GEJ. There was a very good response to chemoradiation (Mandard Tumour Regression Grade 3). Esophageal and gastric resection margins were clear of viable tumor, and two of 26 lymph nodes contained small deposits of adenocarcinoma. Furthermore, multiple coeliac lymph nodes, as well as the No 12 lymph node, showed no evidence of metastatic disease. Over 90% of the tumor was necrotic as a consequence of the chemoradiation and no evidence of malignancy was identified in the liver or the spleen. 

The postoperative course was uneventful, and the patient was discharged 13 days postoperatively. No further radiotherapy treatment was deemed necessary. In the following months, he received four cycles of DCF (docetaxel, cisplatin, and 5FU) chemotherapy and experienced problems with nutritional support, neutropenic sepsis, diarrhea, and intestinal bacterial overgrowth, all of which were controlled medically. A taxane-based regimen was selected based upon the relatively poor response to neoadjuvant oxaliplatin and capecitabine. Follow-up CT scans and tumor markers were performed at three-month intervals for three years, and at six-month intervals for a further two years. All showed graft patency and no local recurrence or metastatic disease. The patient is alive and well 5.5 years postoperatively with no evidence of recurrent disease; his weight is stable, and he returned to work and normal activities of daily life six months postoperatively.

## Discussion

Currently, the Union for International Cancer Control (UICC) TNM classification is the main tool for defining the operability of a cancer patient, however, only from a purely anatomical point of view [4]. The terms unresectable and inoperable are often used without actually addressing the correct meaning of each term. By labeling a patient as ‘inoperable’, the emphasis is not placed on the technical challenge, as that could perhaps be surmounted, but on the futility of the procedure based on past surgical experience and published outcome data. In this case, an optimal response to neoadjuvant therapy and extended period of surveillance suggested an indolent tumor biology in a fit young patient with no comorbidities; thus, there is a small group of patients where the futility arguments are not necessarily valid.

Although the TNM classification is of value in determining the extent of a tumor and is therefore a guide to resectability, it does not take into account the ‘immune score’; namely, the role of adaptive and innate immunity in determining the tumor microenvironment [5]. There is increasing evidence that the immune profile of a tumor has profound effects on its behavior and that a coordinated adaptive immune reaction may well predict clinical outcome more accurately than tumor staging [6].

There is no clear definition of standard therapy, but in the UK, it usually consists of perioperative chemotherapy and surgical resection based upon the MAGIC trial data, with chemoradiotherapy usually added postoperatively for adverse histopathologic features and patients with a high-risk of local recurrence (e.g. positive circumferential margins) [2, 7]. Increasing trimodal therapy consisting of neoadjuvant chemoradiotherapy prior to surgical resection is used, based upon the Dutch CROSS trial.

Another important factor in defining operability and resectability is the encasement of major vascular structures, such as the coeliac, common hepatic, or splenic arteries. En bloc resection of the coeliac trunk, along with the coeliac nervous plexus and lymph nodes for advanced gastric cancer, has been technically possible over the last 60 years, and several revascularization strategies have been subsequently described [3, 8]. However, outcome data has historically been poor, and this may largely be due to poor patient selection. 

The value of pathologic response to neoadjuvant therapy in different types of cancer has been investigated. In particular, pathologic response in patients with esophageal cancer, who received preoperative chemotherapy or chemoradiation, was shown to play an important role in predicting survival outcomes. However, there is a need for additional surrogate markers for reliably predicting the biological behavior of the tumor. Recent and ongoing developments in proteomics, miRNA, and cDNA microarray technology may well have a role to play in the future in accurately determining therapeutic responses, whilst more sensitive and detailed molecular analysis of pathologic and immunologic responses could assist in predicting survival more accurately.

It has been shown that downstaging of tumors can be achieved with neoadjuvant therapy, i.e., initially unresectable colorectal liver metastases [9]. In the case of gastroesophageal cancer patients, the combination of the clinicopathologic features, imaging, and molecular biomarkers facilitates the establishment of a predictive value for neoadjuvant therapy. Such biomarkers previously reported include the NF-κB, the EGFR family, and VEGF, as well as gene expression profiling. Extensive research is also being conducted into the characterization of ‘immunological landscapes’ to predict tumor behavior and international collaborative studies are addressing the development of an ‘immunoscore’ for the same purpose [10]. The ultimate goal would be to develop techniques to assess immunoscores on very small biopsies or on samples of peripheral blood.

As regards to our patient, the tumor was aggressive in terms of its local behaviour but less so in terms of metastatic potential, with the patient having stable disease a year following initial diagnosis and with this being a key feature in the disease being considered potentially operable.

In this case report, we address the complex issue of inoperability versus unresectability, as well as the important issue of patient selection for a multi-modality “off protocol” approach to complex tumors. Biological behavior and response of a tumor to chemotherapy and/or chemoradiation, along with the ability of the surgical team to undertake a potentially curative resection, even when technically very challenging, should all be taken into consideration. The word ‘inoperable’ should be used only in instances in which surgery could likely lead to the "on the table" death of the patient or to unacceptable postoperative morbidity or mortality. In other cases, they should be considered “potentially resectable” with the question being asked whether surgery would confer a clinical advantage and what potential benefits could justify the risk involved.

## Conclusions

Previously, surgical resection has been determined by tumor size and location, lymph node involvement, and the presence of metastatic disease. Although useful in actuarial terms, this is far from individualization in the world of “personalized medicine”. The personal selection by tumor biological behavior and response to neoadjuvant therapy, in addition to these other factors, would be more sophisticated and take into account additional factors, such as comorbidities, performance status, and immunology.
